# Tribocatalytically-activated formation of protective friction and wear reducing carbon coatings from alkane environment

**DOI:** 10.1038/s41598-021-00044-9

**Published:** 2021-10-19

**Authors:** Asghar Shirani, Yuzhe Li, Osman Levent Eryilmaz, Diana Berman

**Affiliations:** 1 Department of Materials Science and Engineering, University of North Texas, Denton, TX 76203 USA; 2 Applied Materials Division, Argonne National Laboratory, Lemont, IL 60439 USA

**Keywords:** Materials for energy and catalysis, Nanoscale materials, Structural materials

## Abstract

Minimizing the wear of the surfaces exposed to mechanical shear stresses is a critical challenge for maximizing the lifespan of rotary mechanical parts. In this study, we have discovered the anti-wear capability of a series of metal nitride-copper nanocomposite coatings tested in a liquid hydrocarbon environment. The results indicate substantial reduction of the wear in comparison to the uncoated steel substrate. Analysis of the wear tracks indicates the formation of carbon-based protective films directly at the sliding interface during the tribological tests. Raman spectroscopy mapping of the wear track suggests the amorphous carbon (a-C) nature of the formed tribofilm. Further analysis of the tribocatalytic activity of the best coating candidate, MoN-Cu, as a function of load (0.25–1 N) and temperature (25 °C and 50 °C) was performed in three alkane solutions, decane, dodecane, and hexadecane. Results indicated that elevated temperature and high contact pressure lead to different tribological characteristics of the coating tested in different environments. The elemental energy dispersive x-ray spectroscopy analysis and Raman analysis revealed formation of the amorphous carbon film that facilitates easy shearing at the contact interface thus enabling more stable friction behavior and lower wear of the tribocatalytic coating. These findings provide new insights into the tribocatalysis mechanism that enables the formation of zero-wear coatings.

## Introduction

Sliding, rolling, or rotating contact interfaces in every manmade, natural or biological system generate friction^1,2^. If not reduced or controlled effectively, high friction often leads to higher wear losses and, hence, shorter life and poor reliability^3^. Consequently, friction has been one of the most active fields of study^4,5^. Many researchers are still working to understand the root causes of friction and new ways to nearly eliminate it to achieve much higher efficiency and longer durability in all types of moving mechanical systems^1,6,7^. While traditional liquid lubricants reduce friction and wear of sliding components, exposure of the system to the boundary lubrication regime under the high contact load may lead to failure of the solid material surfaces^8,9^.

A wide range of solutions, either employing additive packages^10,11^ or pre-coating the surfaces with wear-resistant films^12,13^, has been explored for improving the reliability of the sliding lubricated interfaces. Among the various tribologically-efficient materials, carbon-based films, such as graphene^10,14^, nanodiamonds^15,16^, and diamond-like carbon (DLC) films^17,18^, are of high interest due to their excellent friction and wear reduction potential for applications in various mechanical systems involving both dry^19,20^ and lubricated contacts^21^.

While the introduction of coatings significantly increases the lifetime of the components, the coatings also eventually fail^22^. Recently, the concept of self-replenishing coatings has been evaluated in synthetic oils^23–25^. Erdemir et al.^23^ showed that metal nitride (MeN)-Cu-based materials allowed to facilitate the formation of protective carbon-rich films, DLC, directly at the sliding interfaces. The modelling efforts confirmed by the experimental results indicated that formation of the tribofilms was activated by presence of catalytically-reactive copper clusters exposed to the hydrocarbon source at the contact interfaces during sliding. A similar concept has also been explored for the tribocatalytically-driven coating formation on copper and platinum-containing surfaces from the gas environment^26,27^ thus demonstrating great adaptability potential of the tribocatalysis mechanism.

Here we evaluate the potential of the MeN-Cu-based tribocatalytic coatings to work in an alkane environment which is interesting both from the fundamental point and from the relevance of tribocatalysis for fuel-based lubrication of sliding components in combustion engines^28^. Specifically, we select the most promising candidate among three tribocatalytic coatings with different carrier matrices and probe its tribological characteristics in decane, dodecane, and hexadecane solutions. While synthetic oils impose interest from the tribological perspective, their controlled analysis is complicated by complex chemistry and the presence of additives. We evaluate the effect of the hydrocarbon chemistry on the potential of the tribocatalytic coatings to facilitate the formation of carbon-rich protective films. Our results show significant improvement of the material performance and even allow the material build-up in the contact area thus reversing damage of the materials. Tribofilms formed at the contact interface under high contact pressures and shear during the sliding process can have a significant impact on improving a system’s tribological performance by reducing friction and suppressing the wear of the surfaces.

## Results and discussion

**Figure 1:**
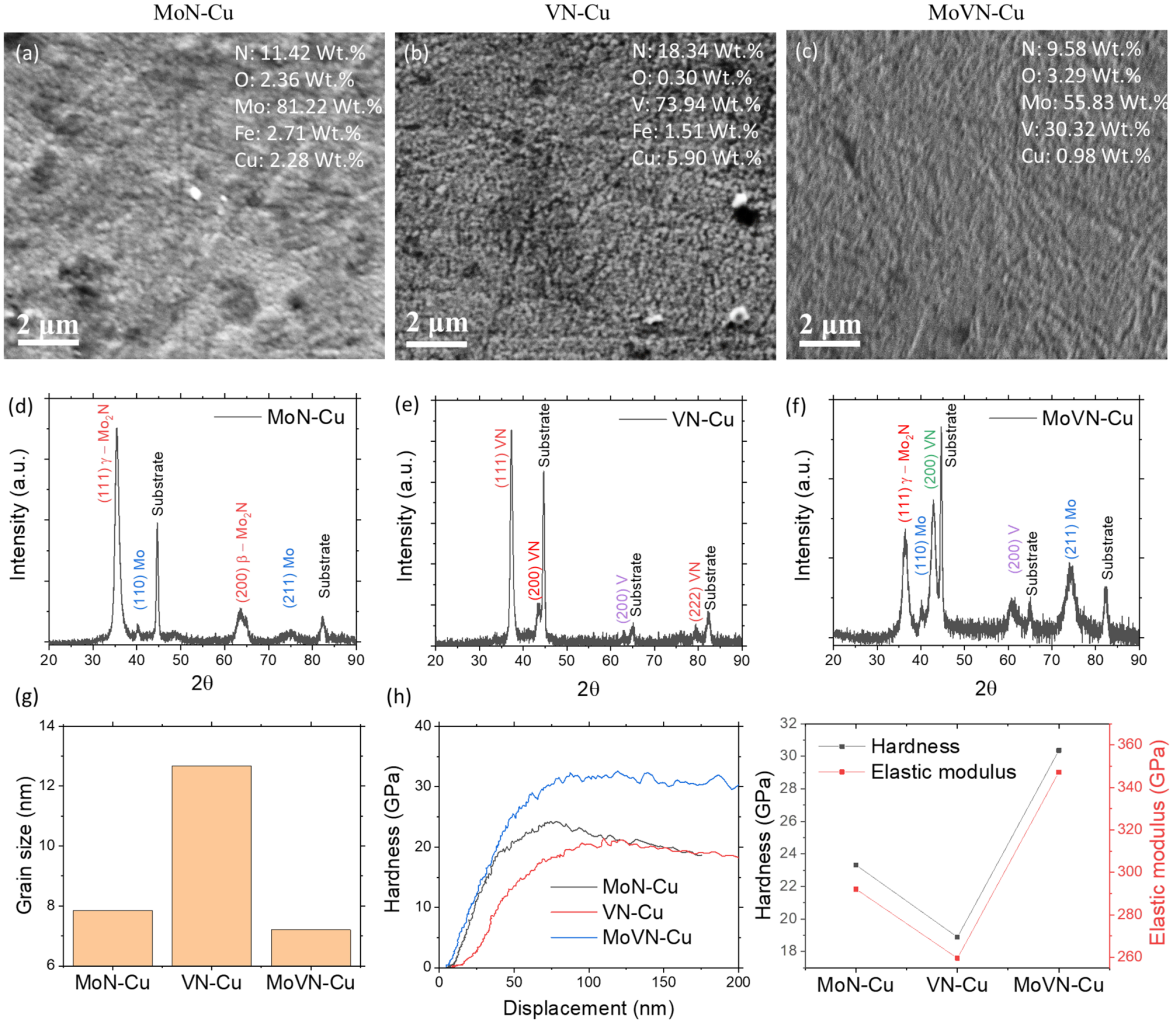
Surface morphology and EDS elemental composition analysis of (**a**) MoN-Cu, (**b**) VN-Cu, and (**c**) MoVN-Cu films. XRD analysis of the (**d**) MoN-Cu, (**e**) VN-Cu, (**f**) MoVN-Cu films, and the calculated grain size for three coatings (**g**). (**h**) Nanoindentation hardness-displacement plots and extrapolated hardness and elastic modulus for the coatings in this study.

Three sputtered nanocomposite coatings, MoN-Cu, VN-Cu, and MoVN-Cu, were prepared as potential tribocatalytically active materials (Fig. 1). The surface morphology analysis of all three coatings shows a relatively uniform texture of the surfaces (Fig. 1a–c). The amount of the incorporated Cu slightly varied among three coatings with VN-Cu having the highest amount of 5.9 wt.% (Fig. 1b) corresponding to the lowest hardness of 18.3 GPa of the film (Fig. 1g). The XRD analysis reveals MoN-Cu, VN-Cu, and MoVN-Cu have Mo_2_N, VN, and MoVN as the dominant phases, respectively. Also, traces of metallic (110) Mo and (200) V phases at 40.1° and 62.9° have been detected in MoN-Cu and VN-Cu, correspondingly (Fig. 1d–f). The grain sizes of the coatings calculated by Debye–Scherrer’s equation were 7–8 nm for MoN-Cu, 12–13 nm for VN-Cu, and 7–8 nm for MoVN-Cu. The nano-indentation results of the coatings indicate MoVN-Cu having the highest hardness and elastic modulus of 30.3 GPa and 346 GPa, respectively.

**Figure 2:**
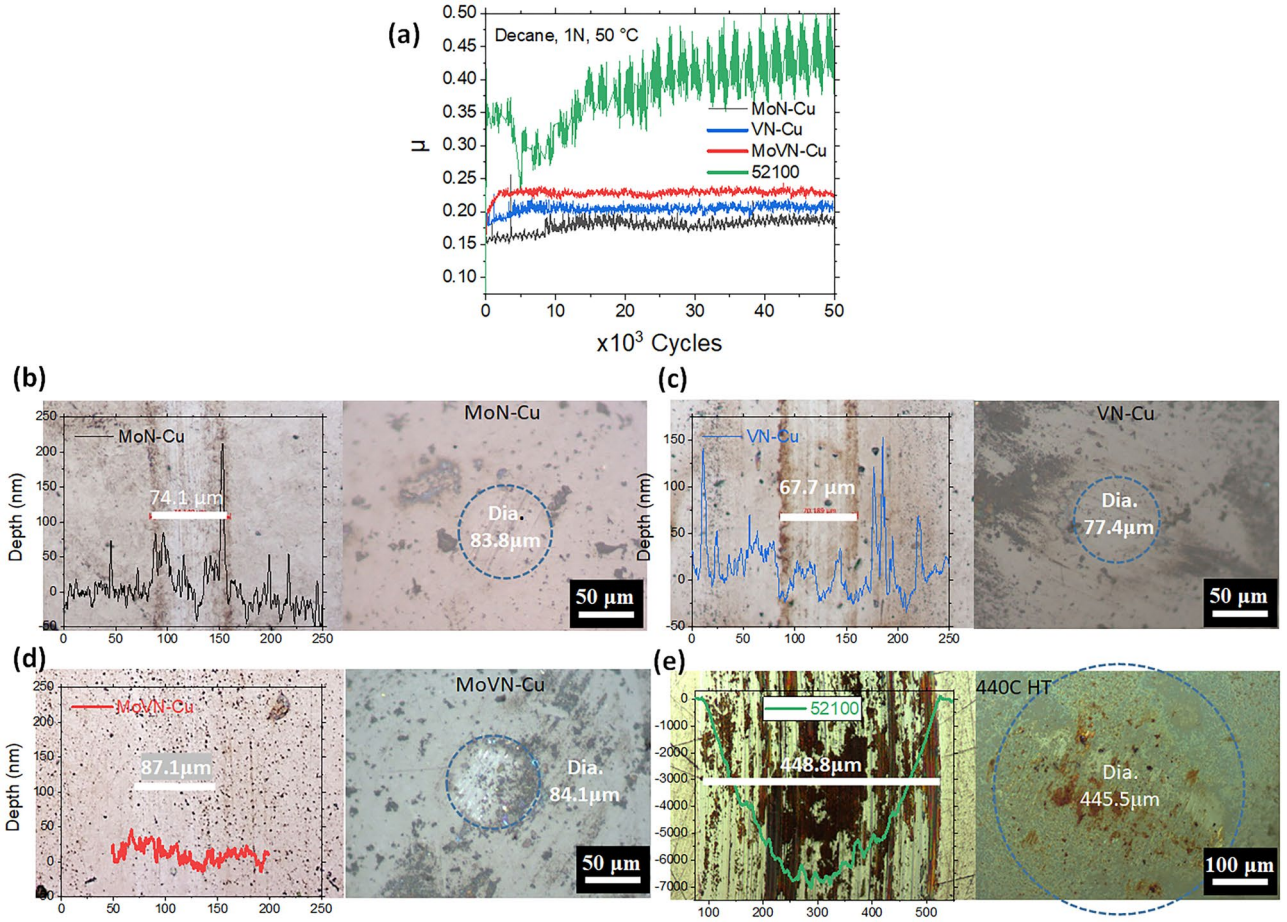
(**a**) Coefficient of friction values for films tested in decane at 1 N load and 50 °C. Optical micrographs of the contact areas and inserted stylus profilometry results of the wear tracks formed on (**b**) MoN-Cu, (c) VN-Cu, (d) MoVN-Cu films, and (e) 52100 steel substrate used as the reference sample.

To determine which coating among the three candidates has the best tribological performance, a set of tribology tests was conducted in decane at 1 N load at 50 °C, followed by the analysis of the wear tracks (Fig. 2). The results were compared to the uncoated steel with 55–60 HRC hardness that was used as a coating substrate material. Among all samples in this study, MoN-Cu nanocomposite demonstrated the lowest coefficient of friction (COF) values. The optical micrographs revealed that all three sputtered nanocomposite coatings have much smaller wear than the uncoated steel. Surprisingly, the stylus profilometry analysis demonstrated that the use of the MoN-Cu coating did not only drastically suppress the wear but even resulted in protective tribofilm material build-up as was indicated by positive cross-section profile changes of the wear track. This film formation for MoN-Cu has been more vivid than for two other samples, VN-Cu and MoVN-Cu. The results suggest that MoN-Cu has the highest tendency to form a protective layer on the contact area that protects the surface from mass loss during tribology (Fig. 2a–e).

**Figure 3:**
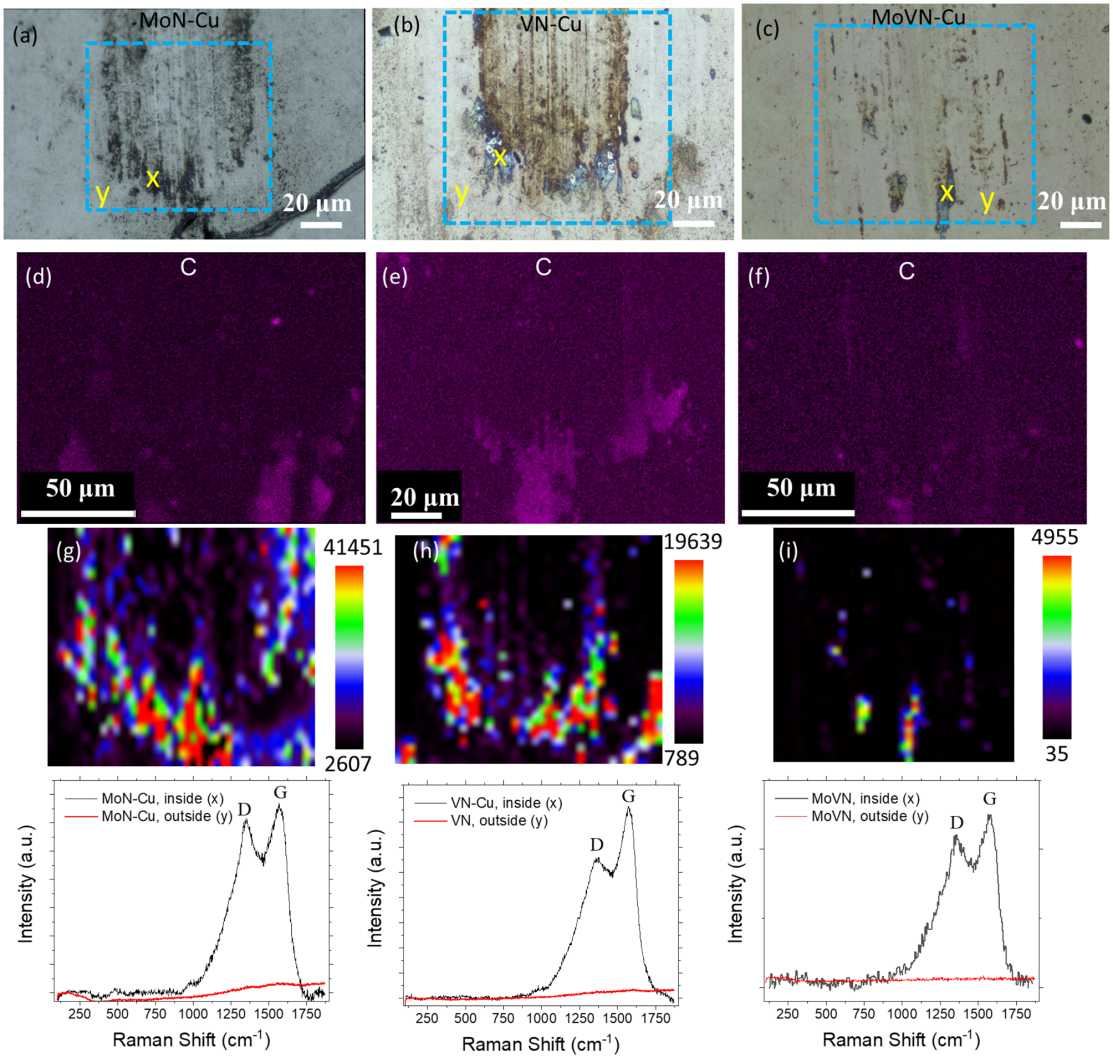
Optical micrographs of the wear tracks, C elemental EDS mapping, and the corresponding Raman mapping for (**a**, **d**, **g**) MoN-Cu, (**b**, **e**, **h**) VN-Cu, and (**c**, **f**, **i**) MoVN-Cu films, respectively. The 2D-Raman analysis is supported by the Raman spectrum collection for two selected spots, inside and outside of the wear track. The tribology tests were performed in decane at 1 N load and 50 °C.

The EDS elemental composition analysis and Raman 2D mapping were performed to identify the nature of the formed during sliding tribofilms (Fig. 3). The blue dashed lines on the optical micrographs indicate the areas for which EDS and Raman analyses were performed. EDS mapping results clearly show the carbon nature of the formed films. Notably, the presence of carbon in the wear tracks is more pronounced for MoN-Cu and VN-Cu samples. 2D Raman analysis indicates the characteristic carbon D (at ~ 1340 cm^-1^) and G peaks (at ~ 1560 cm^-1^) suggesting the Diamond-Like-Carbon (DLC) structure, similarly to the previously observed tribocatalytic formation of DLC from oils^23^. Raman spectroscopy reveals the relatively high intensity of DLC formation in the MoN-Cu wear track which is also supported by higher uniformity of the G-band intensity in the wear track (Fig. 3g). These results suggest that though the hardness is usually considered as a positive contributor to the wear reduction of the coating^9^, it still should be low enough to release the catalytic centers to the hydrocarbon environment. Specifically, the MoVN-Cu sample has the highest hardness among other candidates, 30.3 GPa (Fig. 1g), but the lowest Cu content results in the minimum C formation in the wear track (Fig. 3f). The softest among the three candidates (with the highest Cu content), VN-Cu coating, shows some lowering in friction and more distinguished signs of carbon film formation. Meanwhile, the middle case, MoN-Cu film with ~ 2.3 wt% of Cu, demonstrates the highest promise to lowering the friction and surviving the overall wear of the surfaces by facilitating higher carbon film formation activity as indicated from the characteristic Raman peak intensity.

**Figure 4:**
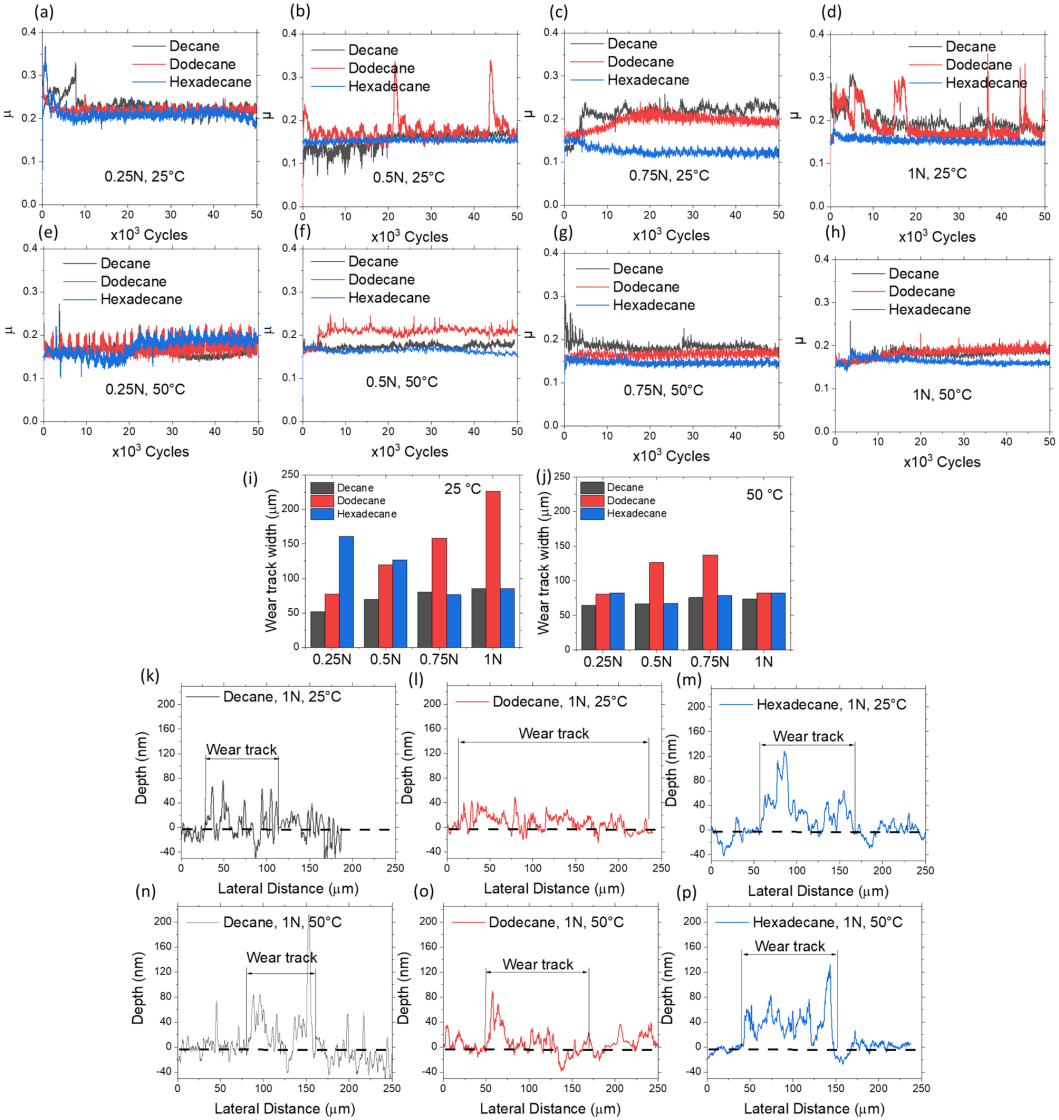
Coefficient of friction behavior of the MoN-Cu coating tribologically tested in decane, dodecane, and hexadecane at 25 and 50 °C at applied loads of (**a**, **e**) 0.25 N, (b, f) 0.5 N, (**c**, **g**) 0.75 N, and (**d**, **h**) 1 N, respectively. Width of the wear tracks formed on MoN-Cu coatings at (**i**) 25 °C and (**j**) 50 °C as load progresses within the 0.25–1 N range. Stylus profilometry results showing film formation in the wear track during the tribology tests in decane (**k**, **n**), dodecane (**l**, **o**), and hexadecane (**m**, **p**) at 25 °C, and 50 °C, respectively.

Since MoN-Cu had the lowest COF (Fig. 2a) and the most promising film formation among other candidates (Fig. 3), it was selected to further understand how load, temperature, and different alkane solutions influence the DLC film formation of the coatings. The results from the tribology tests performed at various loads within the range of 0.25–1 N and at 25 and 50 °C upon immersion in decane, dodecane, and hexadecane are illustrated in Fig. 4a–h. At low temperature and contact pressure, the COF behavior is not steady and results in higher friction values (Fig. 4b,d). As the temperature and load increase, the COF becomes steadier (Fig. 4h). Though the observed average friction changes are not as dramatic, the wear evaluation (Figs. 4i,j) suggests that the increase in the applied load may lead to different outcomes of the tribocatalytic activity of the coatings. While in case of dodecane, increase in contact load leads to higher width of the formed wear track as an indicative of the larger wear, sliding in decane results in almost no effect on the wear. In contrast, hexadecane use leads to the lowering of the wear, as indicated by the reduction of the wear track width. In all three case, there is a competition of two mechanisms working at the same time, formation and replenishment of the carbon-based tribofilm and the friction-induced wear of the materials during the tribotests. Dominancy of one mechanism over the other results in observed changes in the behavior.

The summary of the wear track width changes for each sample after tribology tests at 25 and 50 °C is represented in Figs. 4i,j, respectively. Direct comparison between 25 and 50 °C runs suggests that raising temperature reduces the wear track width, i.e., at 0.25 N in hexadecane, the wear track width was 160.8 µm at 25 °C and reduced to 82.3 µm as temperature increased to 50 °C. Stylus profilometry results indicate that the nature of the hydrocarbon is also important for protective film formation. Interestingly, decane and hexadecane form the carbon film in the wear tracks more readily than dodecane; it is consistent with the film formation across the whole wear track (Fig. 4k–p). This formation is further accelerated at 50 °C, which is supported by the larger volume of carbon built-up in the wear track (Fig. 4n–p). These results are in agreement with the previously reported model for the tribofilm formation from the Zinc Dialkyl Dithiophosphate (ZDDP) additives in oil activated at different temperatures^11^.

**Figure 5:**
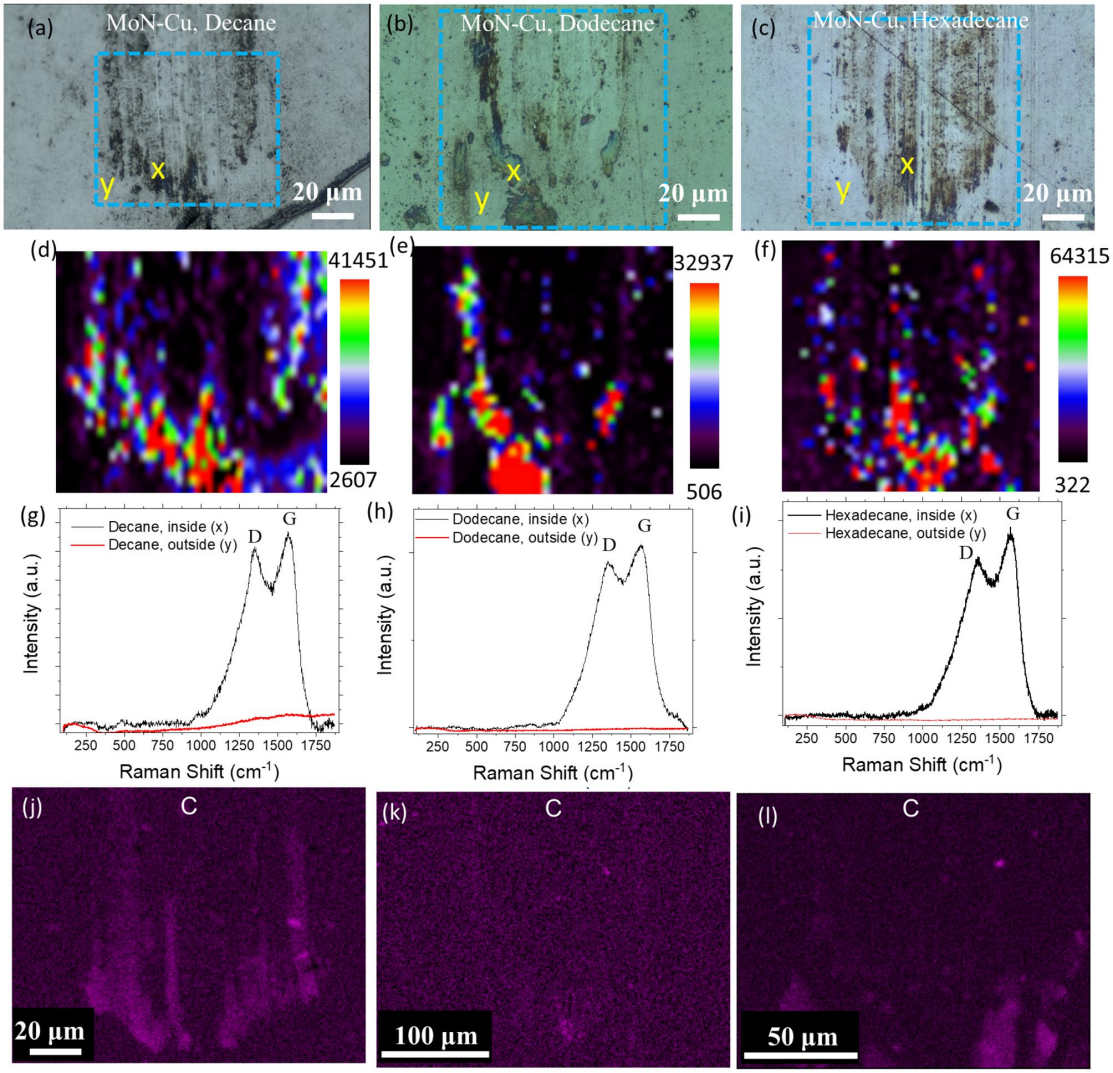
Optical micrographs of the wear tracks, 2D-Raman mapping of carbon G peak (at ~ 1560 cm^-1^) in the area highlighted with a blue dashed line, and Raman spectra for two points inside and outside of the wear tracks formed on MoN-Cu coating in (**a**, **d**, **g**) decane, (**b**, **e**, **h**) dodecane, and (**c**, **f**, **i**) hexadecane solutions, respectively, at 1 N applied load and 50 °C. EDS C elemental mapping of MoN-Cu wear tracks produced during the tribology tests at 1 N and 50 °C in (**j**) decane, (**k**) dodecane, and (**l**) hexadecane.

To have a better knowledge of how alkanes impact the DLC film formation on the MoN-Cu, a series of 2D Raman maps were collected for the wear tracks formed during sliding (Fig. 5). The characteristic DLC phase D and G-bands were observed for the wear tracks formed in all three candidate hydrocarbons. However, the intensity of the G-band for decane and hexadecane was higher than for dodecane (Fig. 5d–f). Also, the distribution of the DLC in the dodecane wear track was less uniform (Fig. 5e). EDS mapping of the wear track reveals the same conclusion, much less carbon is formed from dodecane environment (Fig. 5j–l).

The observed results suggest that in all the cases, the MoN-Cu surface shows very promising tribocatalytic performance. Similarly to previous studies^23^, this tribocatalytic performance is expected to originate from the copper presence in the coating with the possible contribution of Mo^29^ released during sliding. The contact pressure and temperature supported by local asperity heating events^30^ are assisting in the tribocatalytic process by providing enough energy for alkane chain dehydrogenation and dissociation^31^ in presence of the catalyst leading to the release of carbon and formation of the DLC film.

**Figure 6:**
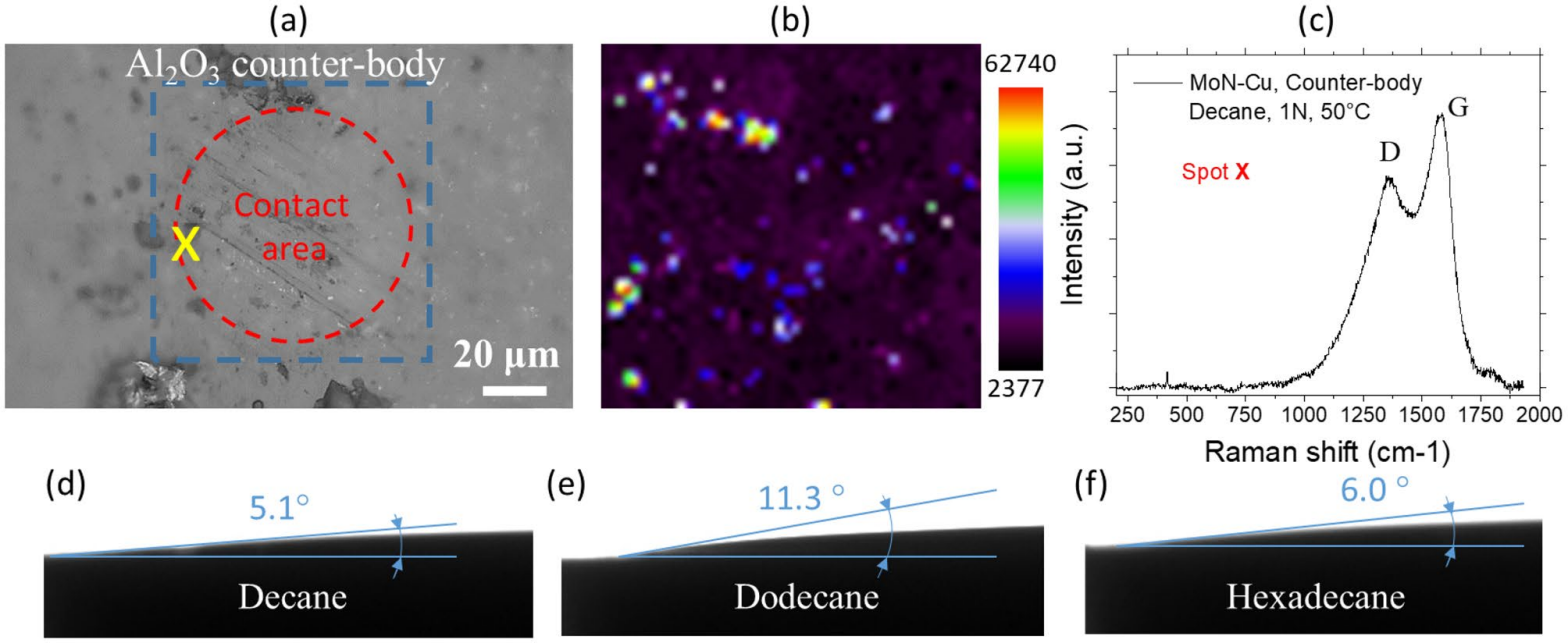
(**a**) Counter-body’s optical micrograph showing the contact area formed on the alumina ball with blue dashed line highlighting the area of interest for the 2D-Raman analysis, (**b**) 2D Raman map of carbon G-band, and (**c**) single Raman spectrum acquired from spot “X” that shows the DLC film transfer on the counter-body. The wetting angle measurements on MoN-Cu surface for (**d**) decane, (**e**) dodecane, and (**f**) hexadecane solutions.

The formed DLC film is expected to transfer on the counterbody to ensure easier shearing and better surface protection from the wear. Indeed, further characterization performed on the alumina counter-body surfaces after the tribology tests revealed the presence of the DLC debris on the counterbody surface (Fig. 6). The 2D-Raman mapping of the alumina surface after the run in decane at 1 N load and 50 °C confirms the DLC tribofilm transfer during sliding (Fig. 6a–c). This transferred film facilities shearing between the nanocomposite surface and Al_2_O_3_ counter-body which is translated into the lower COF and smaller wear track width (Fig. 2a).

Though all three alkanes show the signs of the DLC formation, the rate of the film formation among them differs with hexadecane showing the higher signs of the material build-up, as suggested by profilometry (Fig. 4p) and Raman intensity (Fig. 5f) results, and better coverage of the film in the wear track (Figs. 5c,f). Notably, the contact angle measurement results for three alkanes suggest very good wetting of all three alkanes with a slightly higher contact angle for dodecane than for decane and hexadecane (Fig. 6d–f). This suggests that decane, dodecane, and hexadecane have a high potential for lubricating the surfaces during sliding and thus to form the protective carbon-based tribofilms that can sustain the wear. However, slight reduction in the wetting effectiveness of dodecane may lead to less lubricant supply being available for the surface protection, thus resulting in the observed discrepancies in the tribocatalytic activity.

The long-chain hydrocarbons are generally considered to be more reactive than short-chain hydrocarbons^32^. Previous studies performed on various alkanes inside the diamond anvil cell exposed to high temperature and high-pressure conditions indicated higher yields of carbon deposition from longer chain alkanes^33,34^ that is in agreement with observed better tribocatalytic activity for hexadecane. It should be, however, noted that during the tribosliding, the local temperature increase and pressure distribution are affected by the lubricant presence that can be pushed outside of the sliding contact by the contact pressures^35^ thus leading to boundary lubrication regime of solid/solid contact interface^36,37^. Though the presence of lubricant helps to reduce the wear, as the longer chain hydrocarbons show higher viscosity and thus result in the formation of a thicker lubrication film, less heating in the contact is expected. The competition of the contributions of two interdependent mechanisms, lowering in local heating for longer chain alkanes and reduction in the activation energy for longer chain alkane decomposition, are, therefore, expected to lead to the observed reduced formation of carbon film in dodecane relative to decane and hexadecane.

Formation of the DLC tribofilm plays a pivotal role in observed friction and wear reduction of the coatings in comparison to steel counterparts. When the tribofilm is eventually worn away, the copper clusters in the coating are re-exposed to the hydrocarbons causing the tribocatalysis to restart and develop new layers of tribofilm protecting the surface. The whole process is continuous and self-regulating.

In conclusion, MoN-Cu, MoVN-Cu, and VN-Cu nanocomposite coatings were evaluated for their tribocatalytic potential. These nanocomposites provide relatively high hardness values with the maximum hardness of 30.3 GPa for MoVN-Cu. The tribological behavior of the sputtered nanocomposites coatings tested against alumina counterparts in decane at 1 N load and 50 °C reveals that all three coatings demonstrate large improvement in comparison to the uncoated steel substrate with MoN-Cu having better friction and wear behavior than two other candidates. All three nanocomposite coatings show near-zero wear volume with signs of material built up in the wear track. The wear track characterization reveals the formation of diamond-like carbon film inside the wear track that results in improvement of the tribological performance of the coatings.

Tribology tests on the MoN-Cu sample in the range of 0.25–1 N load and at 25 and 50 °C in decane, dodecane, and hexadecane indicated that the COF behavior becomes steadier and leads to smaller wear track width at the elevated temperature and higher applied load regime.

- Among three selected alkanes, dodecane shows the lowest tribofilm formation tendency. Meanwhile, in the case of decane and hexadecane, the formed tribofilms have a more uniform structure.
- The EDS mapping shows C-rich film formation in the wear track and Raman spectroscopy analysis detects the DLC nature of the tribofilm. Material transfer between the nanocomposite surface and counter-body facilitates easy shearing action.

The differences in the observed hydrocarbon lubrication efficiency and the tribofilm formation activity are attributed to the lower carbon yield from the long chain hydrocarbons and lower wetting of dodecane on the MoN-Cu coating surface.

## Methods

### Sputtering deposition of the coatings

The samples were deposited by direct current magnetron sputtering in a cryo-pumped high vacuum system (the base pressure of ~ 10^–7^ Torr). The samples were Ar ion etched prior to the deposition to eliminate the presence of the adventitious carbon. Pure Mo (99.95%), V (99.95%), and Cu (99.999%) targets were used as the material sources to sputter MoN-Cu, VN-Cu, and MoVN-Cu. The sputtering applied power values were 9 W/cm^2^ for Mo and V sources and 0.45 W/cm^2^ for the Cu source. During the sputtering deposition, the heat-treated AISI 52100 steel substrate was kept at 270 °C. The Ar/N2 ratio was 130 sccm to 55 sccm at 0.4 Pa total pressure. More details on the deposition process can be found in the prior work^27^.

### Tribological tests

The tribological performance testing of the samples was performed using the pin-on-disk tribometer (Anton Paar, TRB^3^) equipped with a 200 ml liquid cell and a heating stage. The 6.35 mm diameter Al_2_O_3_ balls with the initial roughness Ra of ~ 20 nm were used as the counter-bodies. The experiments were performed at two controlled temperatures: 25 and 50 °C with ± 1 °C temperature deviation in three alkane solutions, decane, dodecane, and hexadecane. The tribology tests were done in reciprocating mode at 2 Hz using a 1.4 mm total stroke length. The applied pressure was adjusted within 0.4–0.7 GPa Hertzian contact pressure by alteration the load within the 0.25 to 1 N range. The maximum linear speed was 0.44 mm/s.

### Characterization

Chemical analysis, elemental mapping, and cross-sectional microscopy were conducted using the FEI Quanta 200 SEM equipped with energy-dispersive x-ray spectroscopy (EDS). The composition of the coatings and corresponding phases were tested using the Rigaku Ultima III X-ray diffractometer (XRD) with Cu Kα X-ray source operated with 1°/min scanning rate and 0.02° step increments in θ–2θ scanning mode. The crystallinity of the samples from the XRD spectrum of the coatings has been analyzed using Debye–Scherrer’s equation^38^:

1 $$d=\frac{K\lambda }{\beta \mathrm{cos}\theta }$$

where d is the mean size of the crystalline domain, K is a form factor (here commonly used 0.9), λ is X-ray wavelength (here 1.54 Angstroms), β is the line broadening at half the maximum of the selected XRD peak after subtracting the instrumental peak broadening, θ is the Bragg angle.

Coating roughness and wear track depth profile analysis were performed using the Veeco Dektak 150 stylus profilometer with a 2.5 μm tip radius. The wetting angle values for the alkane solutions on the MoN-Cu surface were separately measured by the sessile drop method using a Ramé-hart 250 contact angle goniometer. The contact angle data were averaged based on five measurements for each solution. The Raman spectroscopy and 2D-Raman mapping were performed by a Renishaw Raman Microscope with a green laser (532 nm wavelength). The nano-indentation measurements were performed by the MTS Nanoindenter XP equipped with Continuous Stiffness Measurement (CSM). 150 nm of the indent’s displacements were used to calibrate and report the hardness values for the coatings.

## Data Availability

The authors declare that data supporting the findings of this study are available within the article files.
